# Supplementary material for: Safety of Administering Live Vaccines during Pregnancy: A Systematic Review and Meta-Analysis of Pregnancy Outcomes
Source: Vaccines (Basel). 2020 Mar 11;8(1):124. doi: 10.3390/vaccines8010124 (PMC7157743; doi:10.3390/vaccines8010124)
Supplement: Supplementary file 1 [file vaccines-08-00124-s001.pdf]

vaccines

Safety of Administering Live Vaccines during Pregnancy: A Systematic Review and Meta-Analysis of Pregnancy Outcomes

Supplementary materials

Table S1. Detailed search strategy using MEDLINE and EMBASE databases - via OVID SP (Wolters Kluwer, 2019).

#	Searches
1	Vaccines, attenuated/ or dengue vaccines/ or ebola vaccines/ or smallpox vaccine/ or japanese encephalitis vaccines/ or exp measles vaccine/ or exp mumps vaccine/ or exp poliovirus vaccine, oral/ or respiratory syncytial virus vaccines/ or rotavirus vaccines/ or exp rubella vaccine/ or yellow fever vaccine/
2	((live or attenuated or measles or mumps or rubella or varicella or yellow fever or dengue or smallpox or japanese encephalitis or JE or ebola or zika or rsv or respiratory syncytial virus or rotavirus or polio) adj3 vaccin*) or mmr or sabin). mp. [mp=title, abstract, original title, name of substance word, subject heading word, floating sub-heading word, keyword heading word, organism supplementary concept word, protocol supplementary concept word, rare disease supplementary concept word, unique identifier, synonyms]
3	exp Pregnancy/ OR exp Pregnant Women/
4	pregnan* or gravid.mp. [mp=title, abstract, original title, name of substance word, subject heading word, floating sub-heading word, keyword heading word, organism supplementary concept word, protocol supplementary concept word, rare disease supplementary concept word, unique identifier, synonyms]
5	exp "Drug-Related Side Effects and Adverse Reactions"/ OR exp Pregnancy Outcome/ OR exp Congenital Abnormalities/ OR exp Stillbirth/ OR exp Abortion, Spontaneous/ OR exp Premature Birth/ OR exp Infant, Low Birth Weight/ OR exp fetal death/ or exp perinatal death/
6	safety or adverse event* or side effect* or adverse effect* or adverse pregnancy outcome* or miscarriage* or spontaneous abortion* or stillbirth* or preterm or premature* or low birth weight or small-for-gestational-age or birth defect* or malformation* or congenital abnormalit* or congenital anomal* or congenital infection* or f?etal death* or neonatal death*.mp. [mp=title, abstract, original title, name of substance word, subject heading word, floating sub-heading word, keyword heading word, organism supplementary concept word, protocol supplementary concept word, rare disease supplementary concept word, unique identifier, synonyms]
7	1 or 2
8	3 or 4
9	5 or 6
10	7 and 8 and 9

Table S2. Risk of bias assessment for miscarriage, according to the ROBINS-I tool.

Study	Bias due to confounding	Bias in selection of participants	Bias in classification of interventions	Bias due to deviations from intended interventions	Bias due to missing data	Bias in measurement of outcomes	Bias in selection of the reported results	Overall risk of bias
Bar-Oz 2004	No information	Serious	Moderate	Serious	Low	Low	Moderate	Serious
Bellows 1949	Serious	Serious	Low	No information	Low	Low	Moderate	Serious
Bourke 1964	Serious	Critical	Serious	Low	Low	Low	Moderate	Critical
Ebbin 1973	Serious	Critical	Moderate	No information	Low	Low	Moderate	Critical
Liebeschuetz 1964	Serious	Critical	Serious	No information	No information	Low	Moderate	Critical
Naderi 1975	Serious	Critical	Moderate	No information	Low	Low	Moderate	Critical
Nishioka 1998	Serious	Serious	Serious	Low	Low	Moderate	Moderate	Serious
Ornoy 1993	Serious	Serious	Moderate	No information	No information	Moderate	Moderate	Serious
Skipetrova 2018	Serious	Low	Low	Serious	Low	Low	Moderate	Serious

Table S3. Risk of bias assessment for stillbirth, according to the ROBINS-I tool.

**Study**	**Bias due to confounding**	**Bias in selection of participants**	**Bias in classification of interventions**	**Bias due to deviations from intended interventions**	**Bias due to missing data**	**Bias in measurement of outcomes**	**Bias in selection of the reported results**	**Overall risk of bias**
Abramowitz 1957	Serious	No information	No information	No information	No information	Low	Moderate	Serious
Bellows 1949	Serious	Serious	Low	No information	Low	Low	Moderate	Serious
Bourke 1964	Serious	Serious	Serious	Low	Low	Low	Moderate	Serious
Harjulehto 1994	Serious	Low	Moderate	Low	Low	Low	Moderate	Serious
Liebeschuetz 1964	Serious	Serious	Serious	No information	No information	Low	Moderate	Serious
Naderi 1975	Serious	Serious	Moderate	No information	Low	Low	Moderate	Serious
Namaei 2008	Serious	Serious	Moderate	Low	Low	Low	Moderate	Serious
Saxen 1968	Serious	Moderate	Serious	No information	Moderate	Low	Moderate	Serious
Skipetrova 2018	Serious	Low	Low	Low	Low	Low	Moderate	Serious

Table S4. Risk of bias assessment for congenital anomalies, according to ROBINS-I tool.

Study	Bias due to confounding	Bias in selection of participants	Bias in classification of interventions	Bias due to deviations from intended interventions	Bias due to missing data	Bias in measurement of outcomes	Bias in selection of the reported results	Overall risk of bias
Harjulehto 1994	Serious	Low	Moderate	Low	Low	Moderate	Moderate	Serious
Ornoy 1993	Serious	Moderate	Moderate	No information	No information	Moderate	Moderate	Serious
Bar-Oz 2004	No information	Serious	Moderate	Serious	Moderate	Moderate	Moderate	Serious
Namaei 2008	Serious	Serious	Moderate	Low	Low	Moderate	Moderate	Serious
Abramowitz 1957	Serious	No information	No information	No information	No information	No information	Moderate	Serious
Bellows 1949	Serious	Serious	Low	No information	Low	Moderate	Moderate	Serious
Bourke 1964	Serious	Serious	Serious	Low	Low	Moderate	Moderate	Serious
Greenberg 1949	Serious	Serious	Serious	Low	No information	Moderate	Moderate	Serious
Liebeschuetz 1964	Serious	Serious	Serious	No information	No information	Moderate	Moderate	Serious
Naderi 1975	Serious	Serious	Moderate	No information	Low	No information	Moderate	Serious
Ryan 2008	Serious	Low	Low	No information	Moderate	Low	Moderate	Serious
Saxen 1968	Serious	Moderate	Serious	No information	Moderate	Moderate	Moderate	Serious

Table S5. Risk of bias assessment for preterm birth, according to the ROBINS-I tool.

Study	Bias due to confounding	Bias in selection of participants	Bias in classification of interventions	Bias due to deviations from intended interventions	Bias due to missing data	Bias in measurement of outcomes	Bias in selection of the reported results	Overall risk of bias
Harjulehto 1994	Serious	Low	Moderate	Low	Low	Moderate	Moderate	Serious
Bar-Oz 2004	No information	Serious	Moderate	Serious	Moderate	Moderate	Moderate	Serious
Namaei 2008	Serious	Serious	Moderate	Low	Low	Moderate	Moderate	Serious
Naderi 1975	Serious	Serious	Moderate	No information	Low	Moderate	Moderate	Serious
Ryan 2008	Serious	Low	Low	No information	Moderate	Low	Moderate	Serious

Table S6. Risk of bias assessment for neonatal death, according to ROBINS-I tool.

Study	Bias due to confounding	Bias in selection of participants	Bias in classification of interventions	Bias due to deviations from intended interventions	Bias due to missing data	Bias in measurement of outcomes	Bias in selection of the reported results	Overall risk of bias
Abramowitz 1957	Serious	No information	No information	No information	No information	Low	Moderate	Serious
Bar-Oz 2004	No information	Serious	Moderate	Serious	Low	Low	Moderate	Serious
Bellows 1949	Serious	Serious	Low	No information	Low	Low	Moderate	Serious
Bourke 1964	Serious	Serious	Serious	Low	Low	Low	Moderate	Serious
Harjulehto 1994	Serious	Low	Moderate	Low	Low	Low	Moderate	Serious

Table S7. Uncontrolled cohorts and pregnancy registries evaluating the pregnancy outcomes after maternal immunization with smallpox vaccine.

Report(s)	Setting	Participants	Exposure in 1st trimester	Miscarriage	Stillbirth	Congenital anomalies	Neonatal death
Ryan et al (2008b) [8]	National Smallpox Vaccine in Pregnancy Registry, established in the United States of America in 2003	376 vaccinated women and 381 fetuses (5 twin sets), 94% without history of previous vaccination	289 women (77%) before 4 weeks EGA	37/376 (9·8%)	5/321 (1·6%)	7/249 (2·8%) among infants with follow-up until 12 months of age	0/39
MacArthur et al (1952) [60]	Questionnaires sent to women vaccinated after an outbreak in Scotland (1950)	4827 questionnaires delivered, 3408 replies (71%), 203 women "successfully" vaccinated during pregnancy or <2 weeks before conception	67 women (33%)	11/203 (5·4%) overall, and 11/67 (16·4%) among those exposed in the 1st trimester	6/192 (3·1%) overall, and 5/56 (8·9%) among those exposed in the 1st trimester	1/186 (0·5%) overall, and 1/56 (1·8%) among those exposed in the 1st trimester, as reported by the mother	1/186 (0·5%)
Wentworth et al (1966) [59]	Continuous series of placentae collected from patients delivered at a maternity hospital after a mass vaccination in Wales (1962)	65 vaccinated women, 100% with history of previous vaccination	56 women (86%)	NA	2/65 (3·1%), one with Rh incompatibility	1/63 (1·6%)	NA

EGA = Estimated gestational age, PCR = Polymerase chain reaction, NA = Not applicable, "Successfully vaccinated": development of vaccination reaction.

Table S8. Uncontrolled cohorts and pregnancy registries evaluating the pregnancy outcomes after maternal immunization with rubella vaccine.

Report(s)	Setting	Intervention*	Participants †	Susceptible before vaccination	Miscarriage	Stillbirth	Congenital infection ‡	Congenital rubella syndrome
MMWR (1989)[10], Bart et al (1985)[45], Preblud (1981 and 1985) [46,47], Modlin et al (1976)[48], Fleet et al (1974)[41], Wyll (1971 and 1973) [49,50]	Vaccine in Pregnancy Registry, CDC (1971 to 1988)	Rubella vaccine 3 months before or after conception	1,221 women (538 with Cendehill or HPV-77, 683 with RA 27/3)	421 women (34·5%)	46/1,136 with known outcome overall (4%), and 19/397 (4·8%) among susceptible participants §	NR	6/215 (2·8%) infants of susceptible women and 5/45 (11·1%) infants of mothers with unknown immunity status.	0/502 infants (306 of susceptible women)
Larson et al (1971) [51]	Women referred to National Institutes of Health, U.S., after immunization during pregnancy	Rubella vaccine <30 days before conception or in first trimester	9 women	One woman (11%), rest unknown	0 (therapeutic abortion in 8/9 vases)	0 (therapeutic abortion performed in 8/9 vases)	Viral isolation in 2/9 placentas and 0/5 fetal tissue cultures	0/1 infant
Tookey et al (1991) [42], Sheppard et al (1986) [52]	Rubella Vaccination Pregnancy Study, United Kingdom (1981-1990)	Rubella vaccination (RA 27/3 or Cendehill) < 3 months before conception or during pregnancy	92 women	NR	2/92 (2·2%)	3/90 (3·3%)	NR	0/87 infants

Badilla et al (2007) [53]	Mass vaccination campaign in Costa Rica, prospective cohort	Measles-Rubella vaccine (RA 27/3) < 3 months before conception or during pregnancy	1,191 women	104 women (8·7%)	128/1,191 (10·7%) overall and 10/104 (9·6%) among susceptible participants	14/1,063 (1·3%) overall and 1/94 (1·1%) among susceptible participants	0/1,049 infants	Defects compatible with CRS: 45/1,049 (4·3%) but none confirmed (other etiologies documented, negative IgM, and viral culture)
Sato et al (2011) [54]	Mass vaccination campaign in Sao Paulo, prospective cohort	Measles-Rubella vaccine (RA 27/3) < 30 days before conception or during pregnancy	2,077 women	644 women (31%)	137/2,077 (6·6%) overall and 34/644 (5·3%) among susceptible participants	12/1,940 (0·6%) overall and 2/610 (0·3%) among susceptible participants	27/580 (4·7%) among infants of susceptible women	0/27 among IgM+ infants
Minussi et al (2008) [55]	Mass campaign in Rio Grande do Sul, Brazil, prospective cohort	Measles-Rubella vaccine < 30 days before conception or during pregnancy	171 women	171 women (100%)	19/171 (11·1%)	3/152 (2%)	10/149 (6·7%) infants	0/10 among IgM(+) infants
Soares et al (2011) [56]	Mass vaccination campaign in Brazil, prospective cohort	Measles-Rubella vaccine (RA 27/3) < 30 days before conception or during pregnancy	2,332 women	2,332 women (100%)	103/1,860 with known outcome (5·5%)	14/1,757 (0·8%)	67/1,647 (4·1%) infants	0/67 among IgM(+) infants; 1 case of CRS due to wild virus

Mistchenko et al (2008) [57] (Conference abstract)	Mass vaccination campaign in Buenos Aires, prospective cohort	Measles-Rubella vaccine < 30 days before conception or during pregnancy	232 women	6 women (2·6%), and 7 indeterminate (3%)	NR	NR	1/6 (16·7%) among infants of susceptible women:	2 infants with cardiomyopathy: 1 with rubella virus in urine (no distinction between wild and vaccine virus); the other with IgM (+) but no virus detected by PCR
Pardon et al (2011) [43]	Mass vaccination campaign in Argentina, Prospective cohort	Measles-Rubella vaccine (RA 27/3) < 30 days before conception or during pregnancy	56 women	7 women (100%)	5/56 (8·9%)	NR	0/5 among infants of susceptible women	0/5 among infants of susceptible women
Enders et al (1985) [61]	Surveillance study on accidental vaccination during pregnancy in Stuttgart, Germany	Rubella vaccine (Cendehill and RA 27/3) within 3 months before or after conception	365 women	146 women (40%), 154 unknown	NR (therapeutic abortion in 34 cases)	NR	2/119 (1·7%) among infants of susceptible and unknown immune status participants	0/194 infants (98 of susceptible women)
Hofmann et al (2000) [13]	Women inadvertently vaccinated during pregnancy in Leipzig, Germany	Rubella vaccine (RA 27/3) periconceptional	6 women	6 women (100%)	0/6	0/6	1/6 infants ⁋	0/6

Hamkar 2006 [12]	Mass vaccination campaign in Iran, prospective cohort	Measles-Rubella vaccine (Edmonston - Zagreb, RA 27/3) < 3 months before conception or during pregnancy	617 women	117 women (19%), rest unknown	0/117 susceptible participants	0/117 susceptible participants	5/535 (0·9%) overall and 2/35 (5·7%) among infants of susceptible women	0/535 infants
Ergenoglu et al (2012) [40]	Mass vaccination campaign in Turkey, prospective cohort	Rubella vaccine (RA 27/3) < 30 days before conception or during 1st trimester	17 women	NR	0/17	0/17	0/17 infants	0/17 infants
Allan et al (1973) [38]	Women referred to the Laboratory of Microbiology in Brisbane, Australia, after immunization in pregnancy	Rubella vaccine (Cendehill) during pregnancy or before conception	65 women	7 susceptible (10·8%), rest unknown	3/65 (4·6%), in addition to 36 therapeutic abortions	0/26	Viral isolation from placental tissue of a healthy infant, among 56 cases with viral cultures. The infant had no serological evidence of infection	0/19 infants
Behnaz et al (2007) [39]	Mass campaign in Yazd, Ian (2004), retrospective cohort	Measles-Rubella vaccine (RA 27/3) < 3 months before conception or during pregnancy	437 infants of vaccinated women	NR	NA	2/437 (0·5%)	2/197 (1%) infants	0/430 infants

Da Silva a Sa et al (2011) [58]	Mass vaccination campaign in Rio de Janeiro, Brazil, prospective cohort	Measles-Rubella vaccine (RA 27/3) < 30 days before conception or during pregnancy	1,636 women	217 women	52/1,636 (3·2%) overall and 10/217 (4·6%) among susceptible participants	7/1,584 (0·4%) overall and 2/207 (1%) among susceptible participants	9/1577 (0·6%) overall and 4/204 (2%) among infants of susceptible women	One case of CRS due to wild virus. No vaccine-associated CRS

NR = Not reported, NA = Not applicable, IgM = Immunoglobulin M, HI = Hemmaglutination-inhibition, CDC = Centers for Disease Control and Prevention, US = United States of America, LBW = Low birth weight, PCR = Polymerase chain reaction. * Edmonston-Zagreb (measles), Cendehill (rubella), HPV-77 (rubella), and RA 27/3 (rubella) refer to the viral strains used for vaccination in each population. † Different methods used to ascertain immune/susceptible status of the mother and CRI in the infant (HI, IgM, IgG avidity, viral isolation). ‡ Serological or virological evidence of congenital infection. IgM (+) refers to detectable rubella IgM. § Includes all spontaneous pregnancy losses, including miscarriage and stillbirth. ⁋ First case of fetal infection by vaccine strain confirmed by PCR and sequencing. .

Table S9. Uncontrolled cohorts and pregnancy registries evaluating pregnancy outcomes after maternal immunization with yellow fever vaccine.

Report(s)	Setting	Intervention and participants*	Participants	Miscarriage	Stillbirth	Congenital infection ‡	Congenital anomalies
Cavalcanti et al (2007)[64], Suzano et al (2006) [16]	Mass vaccination campaign in Campinas, Brazil, prospective cohort	Yellow fever vaccine (17DD) during pregnancy or within 15 days of LMP	441 women (mean EGA at vaccination: 5·7 weeks)	11/441 (2·5%)†	3/441 (0·7%)	0/341 infants §	10/304 (3·3%) with major birth defects, and 62/304 with minor dysmorphisms
Nasidi et al (1993) [17]	Mass vaccination campaign after an outbreak in Nigeria, prospective cohort	Yellow fever vaccine (17D) during pregnancy	101 pregnant women (4 in 1st trimester)	1/101 †	NR	0/40 infants	1/40 (suspected Hirschsprung disease)
Tsai et al (1993) [65]	Mass vaccination campaign in Trinidad, retrospective cohort	Yellow fever vaccine (17D and 17DD) during pregnancy	41 infants of vaccinated women	NA	NA	1/41 (clinically healthy infant)	NR
Robert et al (1999) [66]	Prospectively recorded cases of maternal vaccination provided by ENTIS and the Pharmacovigilance Department of Pasteur Merieux Connaught	Yellow fever vaccine during or shortly before pregnancy	74 women, 58 with complete follow-up	7/58 (12·1%)	0/58	NR	2/46 (4·3%) with major birth defects, and 3/46 (6·5%) with minor dysmorphisms

IgM = Immunoglobulin M, IgG = Immunoglobulin G, LBW = Low birth weight, EGA = Estimated gestational age, YF = Yellow fever, RT-PCR = Reverse transcription polymerase chain reaction, OPV = Oral poliovirus, ENTIS = European Network of Teratology Information Services. *17D and 17DD refer to the viral strains used for vaccination in each population. † Miscarriages probably underestimated due to delayed presentation. ‡ Yellow fever IgM in cord blood or infant serum, suggesting congenital infection. § Yellow fever IgG after 12 months of age detected in 1/233 infants with follow-up.

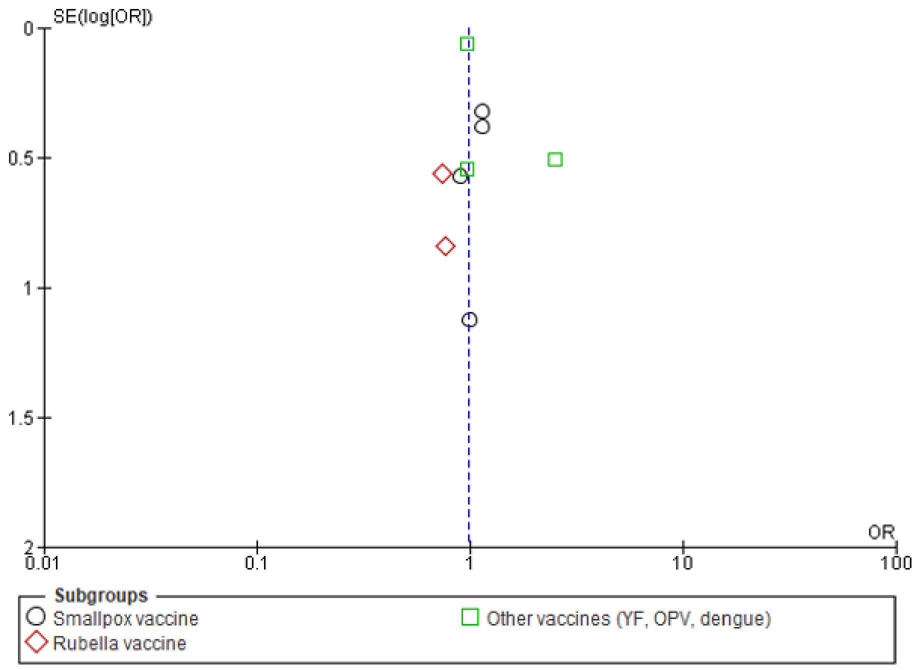

Figure S1. Funnel plot of the studies included in the meta-analysis of the effect of the association of maternal immunization with miscarriage.

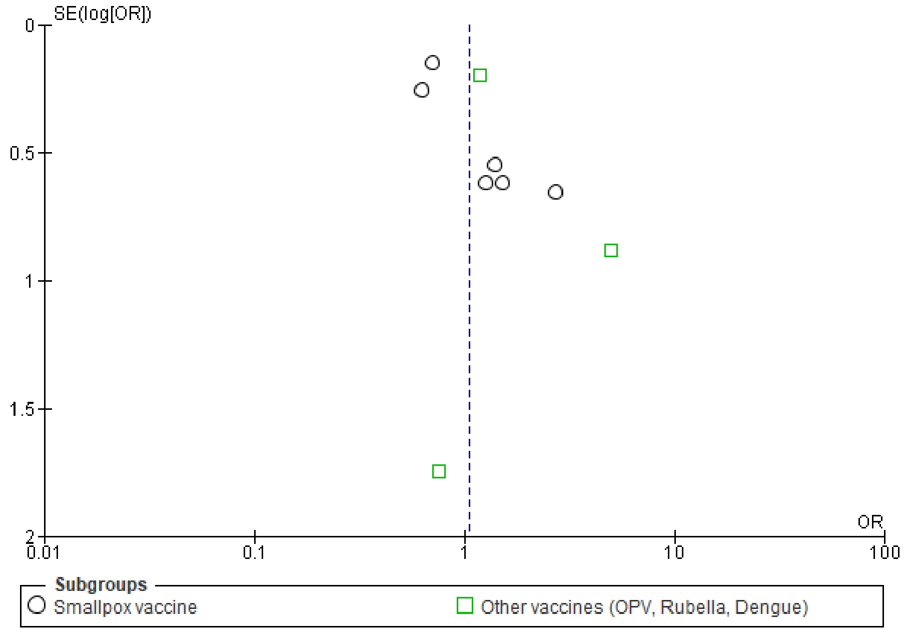

Figure S2. Funnel plot of the studies included in the meta-analysis of the effect of the association between maternal immunization and stillbirth.

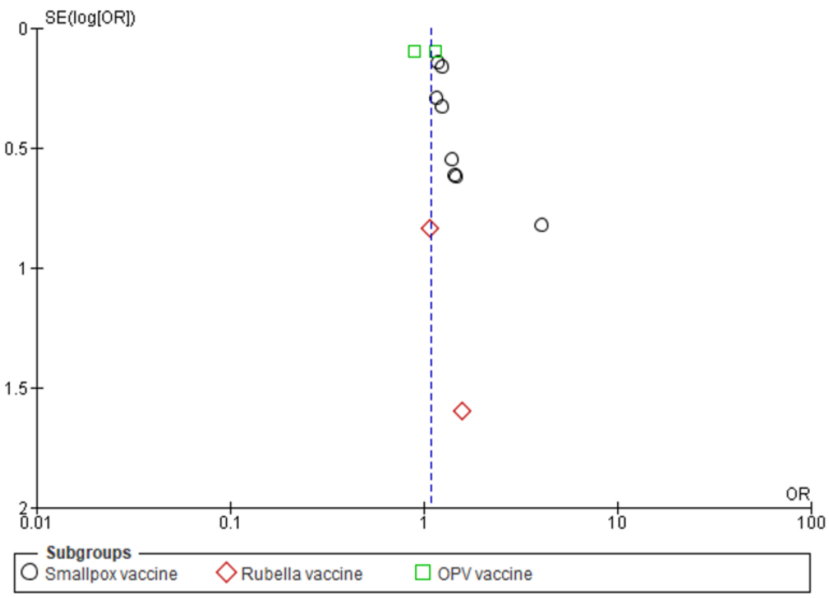

Figure S3. Funnel plot of the studies included in the meta-analysis of the effect of the association between maternal immunization and congenital anomalies.

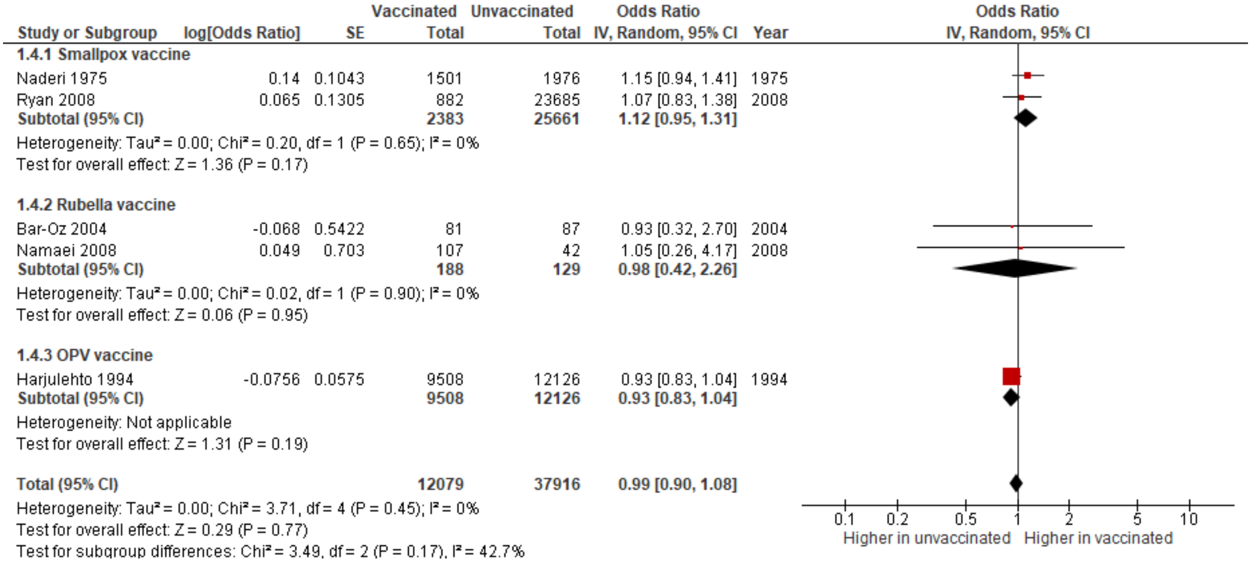

(a)

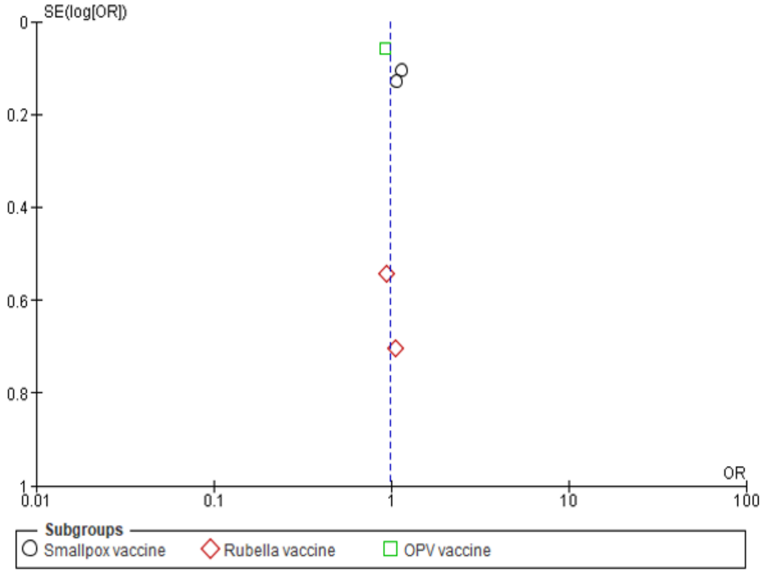

(b)

Figure S4. Meta-analysis of the effect of maternal immunization on preterm birth. Forest plot showing the effect of immunization during pregnancy on the odds of prematurity (**a**), subgrouped by vaccine. Funnel plot showing the effect of vaccination on preterm birth (**b**).

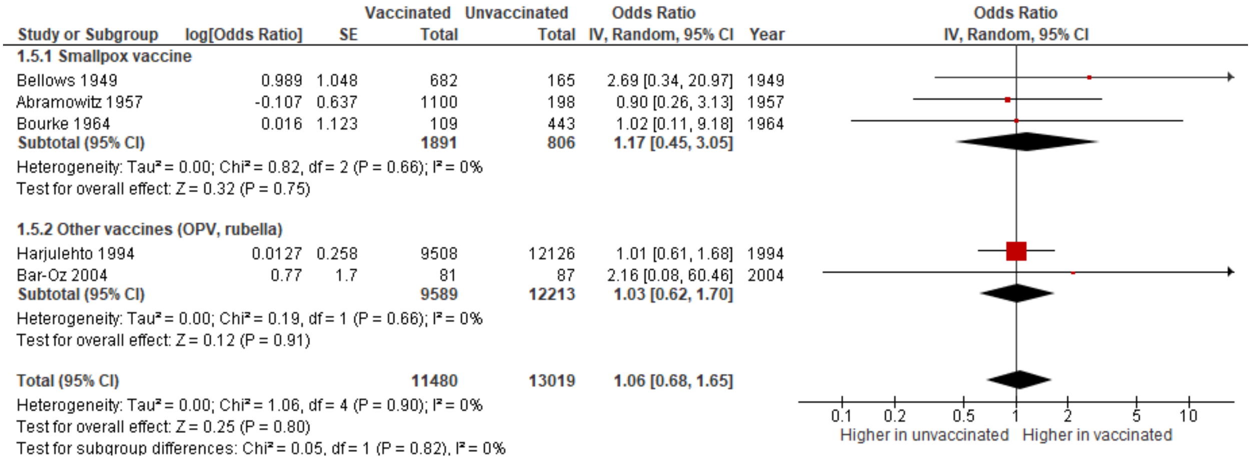

(a)

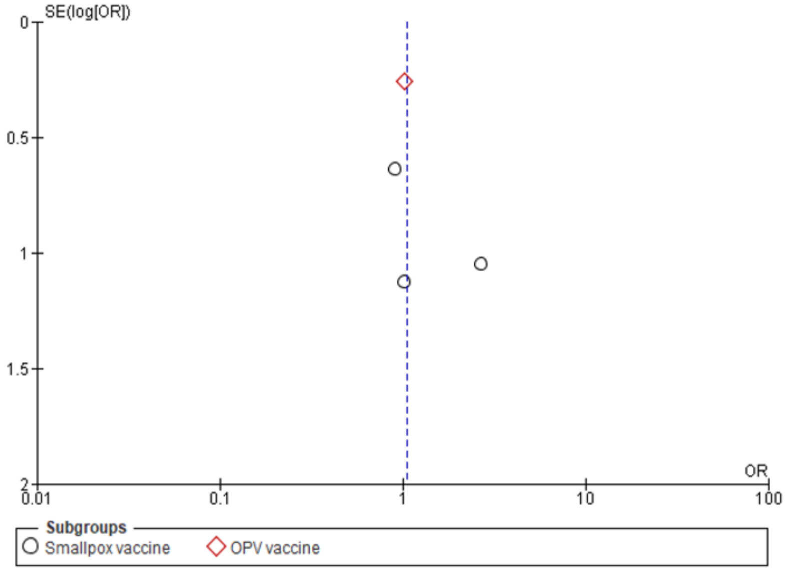

(b)

Figure S5. Meta-analysis of the effect of maternal immunization on neonatal death. Forest plot showing the effect of immunization during pregnancy on the odds of neonatal death (**a**). Funnel plot showing the effect of vaccination on preterm birth (**b**).

© 2020 by the authors. Submitted for possible open access publication under the terms and conditions of the Creative Commons Attribution (CC BY) license (http://creativecommons.org/licenses/by/4.0/).
